# Hybrids of Membrane-Translocating Antimicrobial Peptides Show Enhanced Activity through Membrane Permeabilization

**DOI:** 10.1021/acsmedchemlett.4c00375

**Published:** 2024-10-11

**Authors:** Giulia F. Trevellin, JuYoung Kwag, Michelle L. Shui, Hannah Klim, Valentina Alvarez, Louise E. O. Darling, Donald E. Elmore

**Affiliations:** † Biochemistry Program, Wellesley College, 106 Central St., Wellesley, Massachusetts 02481, United States; ‡ Department of Chemistry, Wellesley College, 106 Central St., Wellesley, Massachusetts 02481, United States; § Department of Biological Sciences, Wellesley College, 106 Central St., Wellesley, Massachusetts 02481, United States

**Keywords:** Histone derived antimicrobial peptide (HDAP), Buforin II, DesHDAP1, Membrane, Translocation, Membrane permeabilization, Hybrid peptide

## Abstract

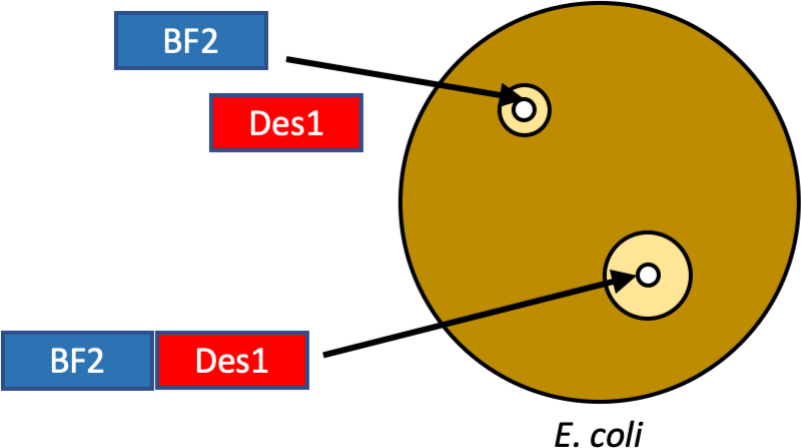

Antimicrobial peptides (AMPs) hold promise as useful tools to combat bacterial infection. Hybrid peptides, made by linking two independent AMPs together through peptide bonds, have the potential for enhancing antimicrobial activity. Here we explore hybrids created by combining two histone-derived antimicrobial peptides (HDAPs), BF2 and DesHDAP1, that each translocate across bacterial membranes. Our work represents the first systematic approach considering the activity and mechanism of hybrids made from two translocating AMPs. BF2/DesHDAP1 hybrids showed increased antimicrobial activity against both Gram-positive and Gram-negative bacteria compared with the parent peptides and no cytotoxicity against eukaryotic cells. Introducing amino acid linkers between the parent peptides did not further enhance the antibacterial activity. The increased antimicrobial activity comes from a mechanistic shift, as hybrid peptides show decreased translocation across bacterial cell membranes but increased membrane permeabilization compared to BF2 and DesHDAP1. These observations lay the groundwork for the further design of hybrid AMPs made from translocating peptides.

Increasing reports of microbial resistance to conventional, small-molecule antibiotics motivates and necessitates work to develop alternative therapies.^1^ Antimicrobial peptides (AMPs) are promising candidates for combating infection and are produced across eukaryotic organisms as well as by prokaryotes as a component of defense or immune systems.^2,3^

The cationic nature of most AMPs promotes electrostatic interactions with overall negatively charged bacterial membranes and drives initial interactions between AMPs and target cells. AMPs preferentially bind bacterial cells over eukaryotes due to the abundance of negatively charged phospholipids in bacterial cell membranes, the negatively charged lipopolysaccharides in the outer membrane of Gram-negative organisms, the acidic polysaccharides in the cell walls of Gram-positive organisms, and the typically more negative membrane potential of prokaryotic cells.^4^

Many AMPs appear to function by compromising the cell membrane to the extent that the leakage of intracellular materials is responsible for bacterial death.^2^ This membrane disruption can occur via different mechanisms, ranging from the formation of systematic pores to more detergent-like wholesale membrane lysis. While membrane-permeabilizing AMPs are more prevalent, a number of other active peptides enter bacterial cells without inducing significant membrane leakage. These translocating AMPs appear to act through targeting essential cytoplasmic functions.^5,6^ While peptides are often categorized as primarily membrane-permeabilizing or primarily translocating, in reality AMP mechanisms can involve a combination of both approaches.^7,8^

One promising approach to engineering more active AMPs is the creation of hybrid peptides, chimeras formed when two parent peptides are linked to create a novel single peptide. This connection can be a simple peptide bond or include additional amino acids as linkers between the two independent parent peptides. However, previous studies have largely focused on hybrids made from membrane-permeabilizing AMPs, such as cecropin, melittin, magainin, cathelicidin, aurein, LL-37, and lactoferricin.^9−23^ While a few recent papers have considered the impact of hybridizing an AMP that primarily induces membrane permeabilization with an AMP that primarily translocates across the membrane,^24,25^ the combination of two membrane-translocating AMPs has not been studied.

Here, we characterize hybrids that combine two histone-derived antimicrobial peptides (HDAPs),^26^ buforin II (BF2) and DesHDAP1, that are identical to sections of histone H2A (Table 1, Supplemental Figure 1). Both BF2 and DesHDAP1 demonstrate a translocating mechanism and are thought to target intracellular nucleic acids.^27−32^ Our data show that hybrids composed of BF2 and DesHDAP1 have enhanced antimicrobial activity against both *Escherichia coli* and *Bacillus subtilis* over the individual parent peptides and beyond mixtures of the two peptides. BF2/DesHDAP1 hybrids also have no appreciable eukaryotic cytotoxicity. We further investigated whether the order of parent peptides or the choice of linker affected the hybrids. While neither of these design criteria affected activity, we did have the surprising result that our hybrids created from two translocating parent peptides consistently showed a shift toward membrane-permeabilizing as their primary mode of antibacterial activity. Moreover, incorporation of a proline linker increased membrane permeabilization.

**Table 1:**
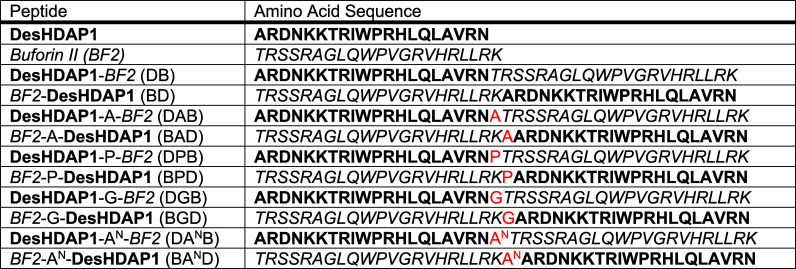
Amino Acid Sequences of the Parent and Hybrid Peptides Used in This Study^a^

a DesHDAP1 portions of sequences are shown in bold, BF2 portions of sequences are shown in italics and linker amino acids are in red text. A^N^ represents hydroxyalanine. BF2 includes an F10W mutation to allow for spectroscopic measurements of the peptide concentration. Physiochemical properties of peptides are provided in Supplemental Table 1.

## DesHDAP1/BF2 Hybrid Peptides Show Enhanced Activity against *E. coli* and *B. subtilis* but No Appreciable Eukaryotic Cytotoxicity

We initially used radial diffusion assays to evaluate the activity of hybrid peptides that combined the sequences of DesHDAP1 and BF2 (Table 1) compared with both the parent peptides and an equimolar mixture of the two peptides (DesHDAP1+BF2). This mixture contained 1 × 10^–4^ M BF2 and 1 × 10^–4^ M DesHDAP1, which is the same total concentration of amino acids as a 1 × 10^–4^ M solution of a hybrid peptide, which includes both the BF2 and DesHDAP1 sequences. This controls for the additional peptide “material” present in a 1 × 10^–4^ M solution of hybrid compared to individual peptides alone at 1 × 10^–4^ M. Equimolar mixtures are defined in an analogous manner for microbroth dilution and cytotoxicity experiments described below.

**Figure 1:**
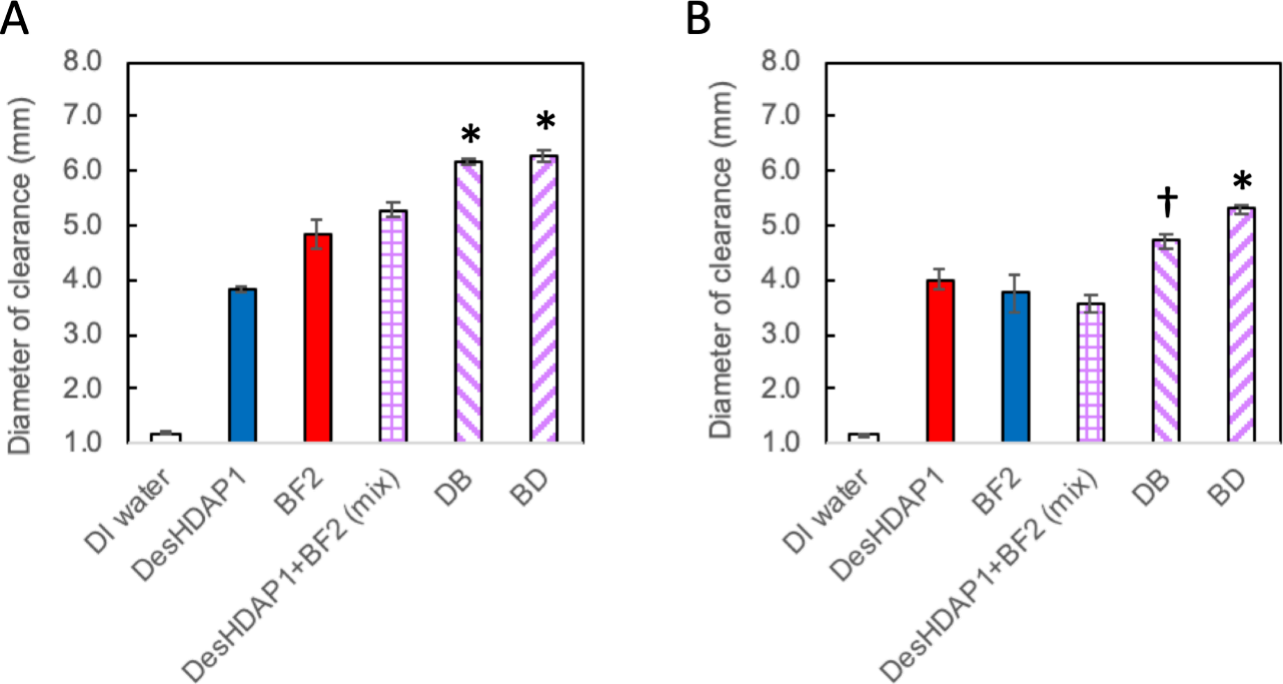
Average diameters of bacterial clearance from radial diffusion assay measurements of parent, mixture, and hybrid antimicrobial peptides with *E. coli* (A) and *B. subtilis* (B). DesHDAP1+BF2 represents an equimolar mixture containing 1 × 10^–4^ M of each of the two parent peptides, the same total amino acid concentration used for hybrid peptides. All averages taken over a minimum of 12 wells from at least three biological replicates. Error bars represent standard error of the mean. * denotes a significant difference (*p* < 0.05) of a hybrid peptide from both parent peptides and the DesHDAP1+BF2 mixture. † denotes a significant difference (*p* < 0.05) of a hybrid peptide from DesHDAP1+BF2 mixture but not parent peptides.

Versions of the hybrid with the DesHDAP1 sequence first (DB) or second (BD) in the peptide both showed significantly increased activity compared to the equimolar mixture (*p* < 0.01) against *E. coli* (Figure 1A) and *B. subtilis* (Figure 1B). No significant differences in antibacterial activity emerged between the DB and the BD forms. This lack of order dependence, as well as the lack of importance for linkers noted below, was interesting given that the C-terminal residues of BF2 are known to be more important than the N-terminal residues for antibacterial activity.^29^ To further confirm the enhanced activity of our DesHDAP1/BF2 hybrid peptides, we also evaluated their activity in microbroth dilution assays (Table 2). Hybrid peptides showed clearly enhanced activity in these experiments with median MIC of 1.1 μM and 4.6 μM for both hybrids in *E. coli* and *B. subtilis*, respectively, compared to an MIC > 18.2 μM for the individual parent peptides or the BF2+DesHDAP1 equimolar mixture in both species.

**Table 2:** Median Minimum Inhibitory Concentrations (MIC) of Parent and Hybrid Antimicrobial Peptides for *E. coli* and *B. subtilis* Measured by Microbroth Dilution Assays^a^

	MIC (μM)	
Peptide	against *E. coli*	against *B. subtilis*
**DesHDAP1**	>18.2	>18.2
*buforin II (BF2)*	>18.2	18.2
**DesHDAP1**+*BF2*	>18.2	>18.2
**DesHDAP1**-*BF2* (DB)	1.1	4.6
*BF2*-**DesHDAP1** (BD)	1.1	4.6
**DesHDAP1**-A-*BF2* (DAB)	1.1	4.6
*BF2*-A-**DesHDAP1** (BAD)	1.1	4.6
**DesHDAP1**-P-*BF2* (DPB)	1.1	4.6
*BF2*-P-**DesHDAP1** (BPD)	1.1	4.6

a DesHDAP1+BF2 represents an equimolar mixture of the two parent peptides, each individually at the reported concentration.

**Figure 2:**
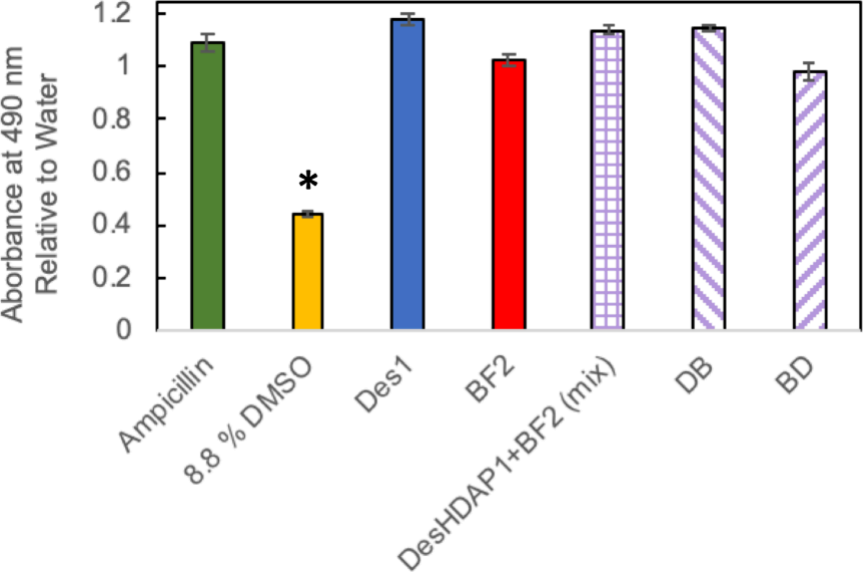
Impact of parent and hybrid peptides on the metabolic activity of HEK 293 cells measured through MTS assays. Metabolic activity is presented as the absorbance at 490 nm relative to that observed for a negative control of cells exposed to water. Ampicillin at 5 mg/mL served as an additional negative control. An 8.8% DMSO solution was used as a control for conditions leading to cell death. DesHDAP1+BF2 represents an equimolar mixture of the two parent peptides each at 18.2 μM. Twenty-four total wells across three biological replicates were averaged for all experimental conditions. Error bars represent standard error of the mean. * denotes conditions with a significantly different (*p* < 0.05) absorbance at 490 nm than water.

We evaluated the potential eukaryotic cytotoxicity of our hybrid peptides by measuring the metabolic activity of HEK 293 cells exposed to peptides in MTS assays. HEK cells were chosen as a tractable and commonly used human cell line that could allow for comparison to other studies. In these experiments, metabolically active cells can enzymatically reduce the MTS reagent into a purple formazan dye that can be spectrophotometrically measured at 490 nm. HEK 293 cells exposed to the hybrid peptides overnight showed no decrease in the formation of formazan relative to cells exposed to water or ampicillin, even at a hybrid peptide concentration at least 4-fold greater than the MIC (Figure 2, Table 2). Thus these peptides (parent, equimolar mixture, and hybrids) do not show cytotoxicity against HEK 239 cells.

## Amino Acid Linkers Have a Minimal Impact on the Antibacterial Activity and Cytotoxicity of DesHDAP1/BF2 Hybrids

Given that the structure of and spacing between parent peptides in a hybrid could alter function, we considered whether the antimicrobial activity of hybrids was impacted by the order of parent peptides and by the inclusion of a linker amino acid to separate the DesHDAP1 and BF2 sequences (Table 1). In addition to utilizing an alanine that would minimally disrupt secondary structure between the two connected peptides we also considered structurally disruptive proline linkers. We chose to focus on the potential role of these disruptive linkers given the role of proline residues in the activity and membrane translocation mechanisms of both BF2^8,27,29^ and DesHDAP1^30^ as well as in designing hybrid AMPs.^9^

**Figure 3:**
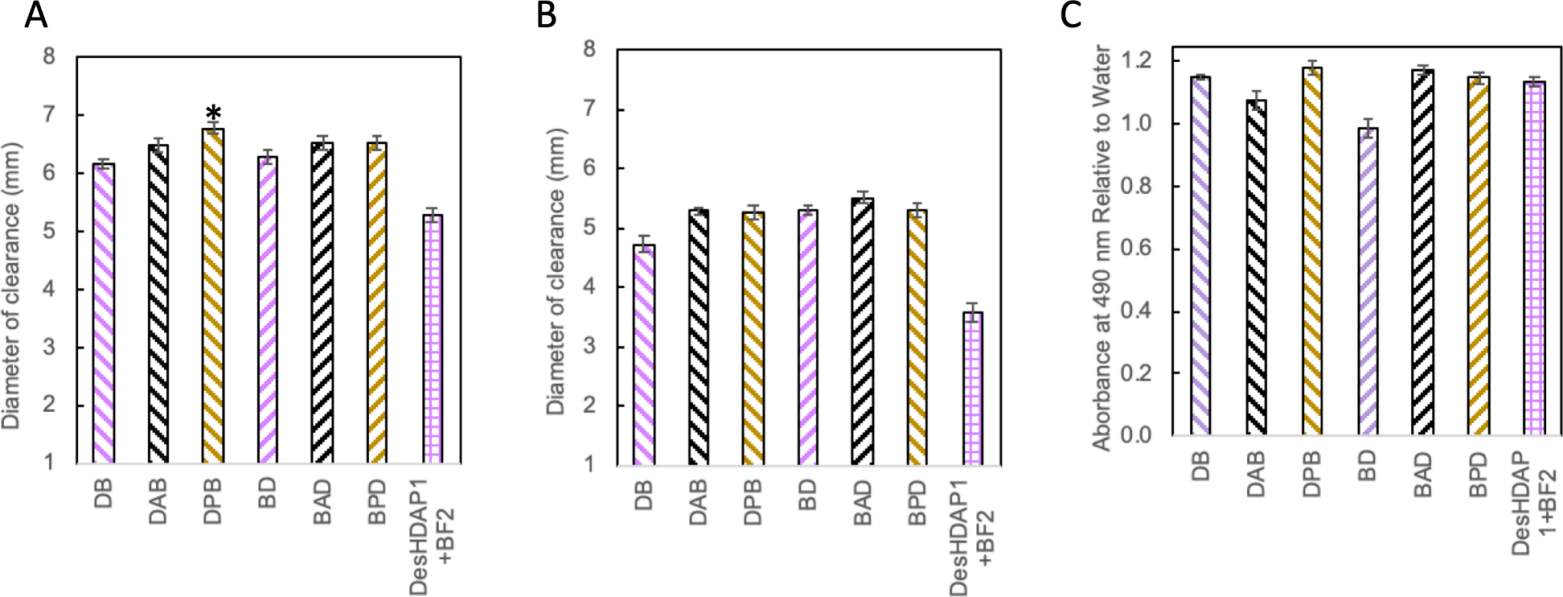
(A, B) Average diameters of bacterial clearance from radial diffusion assay measurements of antibacterial activity against *E. coli* (A) and *B. subtilis* (B) using 1 × 10^–4^ M of hybrid peptides. All averages taken over a minimum of 12 wells from at least 3 biological replicates. Error bars represent standard error of the mean. * denotes a significant difference (*p* < 0.05) of a peptide with a linker from the hybrid without a linker. (C) Impact of hybrid peptides with different linkers on the metabolic activity of HEK cells measured through MTS assays. Metabolic activity is presented as the absorbance at 490 nm relative to that observed for a negative control of cells exposed to water. Cells were exposed to all peptides at 18.2 μM. Twenty-four total wells collected from three biological replicates were averaged for all experimental conditions. Error bars represent standard error of the mean. No peptides showed a significantly decreased absorption from cells exposed to water.

Overall, the effect of different amino acid linkers was minimal in biological activity assays against both bacterial and eukaryotic cells. In radial diffusion assays with *E. coli* (Figure 3A, Supplemental Figure 2A), linkers caused no significant changes in activity for the BD-ordered hybrid. The only statistically significant change in activity for DB hybrids was a slightly increased diameter of clearance with the proline linker in DPB (*p* < 0.01) (Figure 3A). No differences in the MIC were measured for hybrids with different linkers in *E. coli* microbroth dilution assays (Table 2). For *B. subtilis* (Figure 3B), there were no significant differences in activity via radial diffusion assay within either the DB or BD families of peptides with linkers nor were there any changes in MIC values measured in microbroth dilutions (Table 2). Similarly, the incorporation of either alanine or proline linkers did not notably alter the impact of hybrids on eukaryotic cells, with none of the versions leading to significantly decreased metabolic activity relative to cells exposed to water (Figure 3C). These results were similar to our previous work showing minimal effects of inserting a single amino acid linker on the activity of hybrids composed of BF2 or DesHDAP1 with a permeabilizing peptide.^25^

To further explore potential linker impacts, we also considered a structurally “flexible” glycine linker and an *N*-hydroxyalanine linker that would disrupt backbone H-bonding analogous to proline without introducing the steric restrictions of proline (Supplemental Figure 2). However, in radial diffusion assays, hybrids containing glycine or *N*-hydroxyalanine linkers showed no significant differences in activity compared to DB and BD.

## DesHDAP1/BF2 Hybrid Peptides Demonstrate Increased Membrane Permeabilization Compared to Parent Peptides

**Figure 4:**
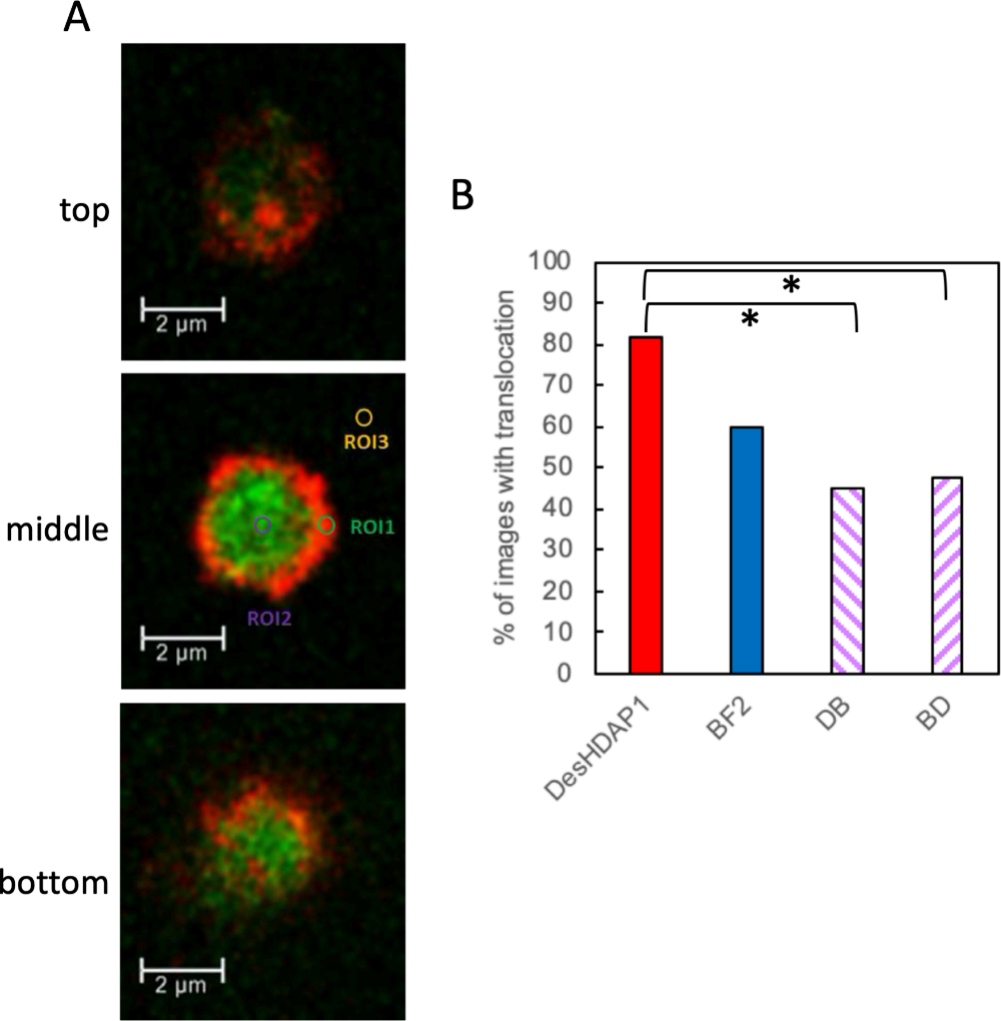
(A) Sample confocal microscopy images collected for an *E. coli* spheroplast exposed to fluorescently labeled DB peptide. Images represent single z-stack slices taken from the top, middle, and bottom of the spheroplast. Green fluorescence was from the FITC-labeled DB peptide, and red fluorescence is from the membrane labeling di-8-ANEPPS. Regions of interest (ROI) used to calculate the translocation ratio as described in the methods are highlighted. (B) Percentage of *E. coli* spheroplasts showing translocation of peptides into the cell using confocal microscopy. Images were collected from at least three separate spheroplast preparations for each peptide with a total of 22 images for DesHDAP1, 20 images for BF2 and DB, and 19 images for BD. * denotes significant difference (*p* < 0.05) for hybrid peptides compared to DesHDAP1.

In previous work, hybrid peptides made by combining DesHDAP1 or BF2 with a membrane-permeabilizing peptide lost the ability to translocate into bacterial cells.^25^ Thus, we hypothesized that hybrids made from DesHDAP1 and BF2, both translocating peptides, would retain their ability to move across the bacterial cell membranes. To that end, we used confocal microscopy to assess whether fluorescently labeled DB and BD hybrid peptides were able to translocate into *E. coli* spheroplasts. Using the larger and spherical spheroplasts allows us to more reliably distinguish between peptides on the surface and on the inside of *E. coli* in confocal images. For all measurements, we collect z-stack slices through spheroplasts to quantitatively compare the fluorescence of peptide on the inside of the cell versus localized on its membrane (Figure 4A). Previous work has shown that these spheroplast measurements correlate well with other cellular studies and can provide useful insight into peptide mechanisms.^7,25^

In our images, both the BD and DB hybrids showed translocation into significantly fewer spheroplasts compared to the DesHDAP1 parent (*p* < 0.05) (Figure 4B). The percentage of images with hybrid translocation was lower than BF2 but not statistically significant (Figure 4B). The ability of hybrids to retain some level of translocation ability was interesting compared to the impacts observed in our work combining DesHDAP1 and BF2 with other membrane-permeabilizing peptides where permeabilizing became the dominant mechanism.^25^

**Figure 5:**
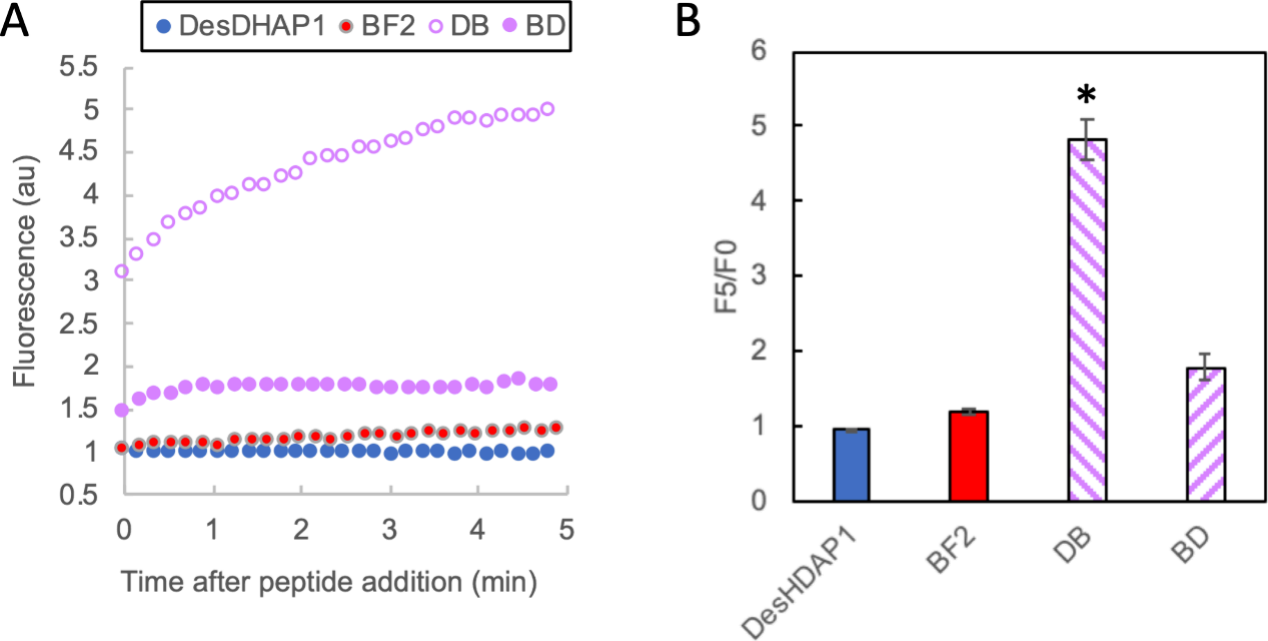
(A) Propidium iodide (PI) fluorescence at 617 nm over time for *E. coli* exposed to 2 μM parent and hybrid peptides. (B) Average F_5_/F_0_ ratio from three experiments, each representing a different overnight culture except for BF2, which was averaged over six experiments from different overnight cultures. Error bars represent standard error of the mean. * denotes a significant difference (*p* < 0.05) of a hybrid peptide from both parent peptides.

We also used propidium iodide (PI) assays to assess whether the DB and BD hybrids induced more bacterial membrane permeabilization than did the parent AMPs (Figure 5). In this experiment, peptides that permeabilize the membrane will lead to enhanced fluorescence as membrane impermeable PI accesses and binds intracellular nucleic acids, increasing fluorescence. BF2 and DesHDAP1 induce minimal permeabilization.^27,30^ However, both BD and DB showed increased PI fluorescence compared to the parent peptides, although this only achieved statistical significance for DB. The particularly large increase may be due to a larger α-helical amphipathic region in DB, discussed below. An increased ability to induce membrane permeabilization may be the source of the enhanced antibacterial activity observed for the hybrid peptides (Figure 1 and Table 2).

## Amino Acid Linkers Impact the Translocation and Permeabilization of BD Hybrids

**Figure 6:**
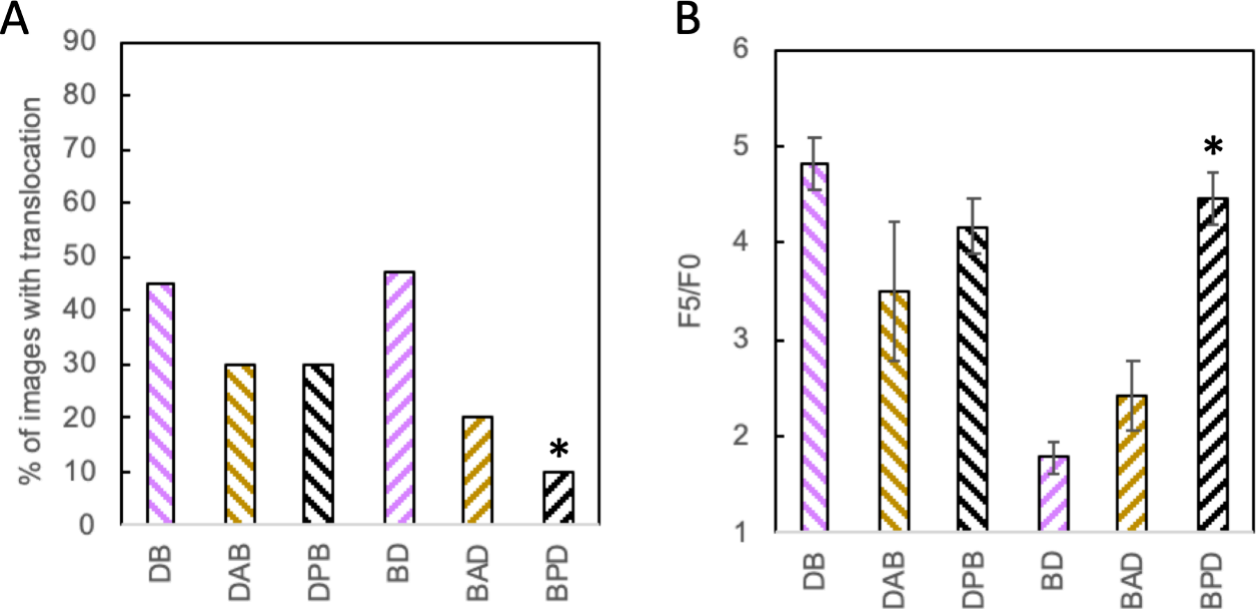
(A) Percentage of *E. coli* spheroplasts showing translocation of peptides into the cell using confocal microscopy (as described in Figure 4). Images were collected from at least three separate spheroplast preparations for each peptide with a total of 19 images for BD and 20 images for all other peptides. * denotes a significant difference (*p* < 0.05) of a peptide with a linker from the hybrid without a linker. (B) Average F_5_/F_0_ ratio from a PI assay in *E. coli* exposed to 2 μM peptide (as described in Figure 5). All values were averaged over three experiments with bacteria from different overnight cultures. Error bars represent standard error of the mean. * denotes a significant difference (*p* < 0.05) of a peptide with a linker from the hybrid without a linker.

Although linkers did not appreciably impact the antimicrobial activity, we considered whether they induced any further mechanistic shifts in DB and BD hybrids. The impact of linkers was quite minimal for DB. Neither DAB nor DPB showed any significant differences in translocation into spheroplasts compared to DB (Figure 6A). They also retained the very high levels of membrane permeabilization seen with the DB (Figure 6B).

However, linkers did impact the membrane translocation and permeabilization behavior of BD. In particular, the proline linker in BPD led to a significant decrease of translocation into spheroplasts (*p* < 0.05) (Figure 6A) and significantly greater membrane permeabilization (*p* < 0.05) compared to BD (Figure 6B). In this case, the enhancement of one mechanism and the relative decrease of another appear to have led to a basically unchanged antimicrobial activity. The translocation and permeabilization of BAD were somewhat intermediate between those of BD and BPD, although neither change emerged as statistically significant at 95% confidence.

**Figure 7:**
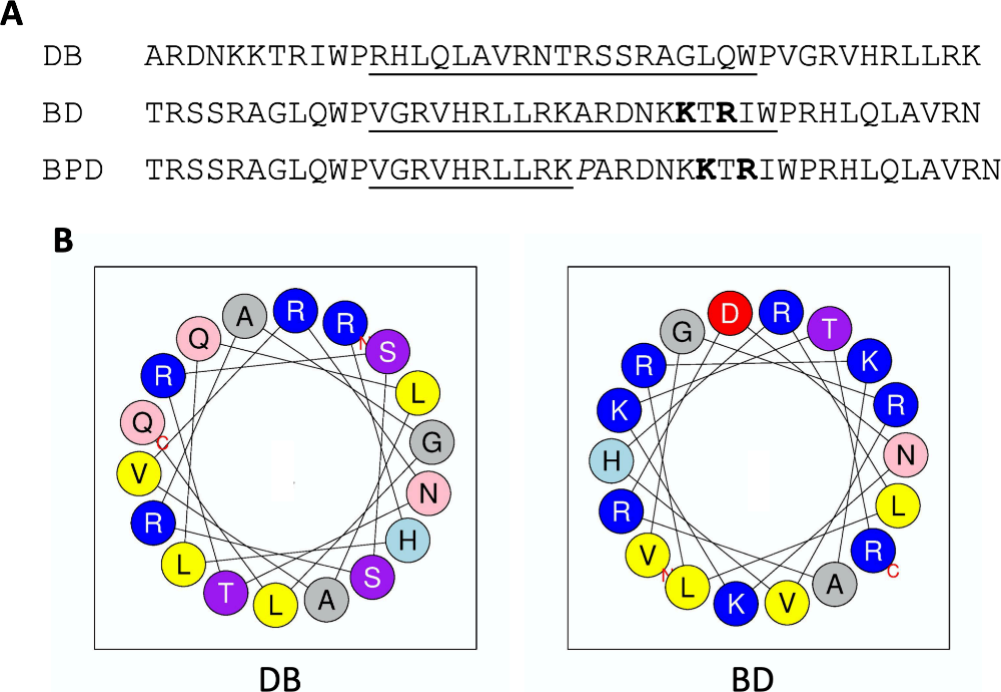
(A) DB and BD sequences with the putative extended α-helical region extending from the end of the first parent peptide to the following proline underlined. The lysine and arginine residues of BD predicted to lie on a hydrophobic helical face are shown in bold. (B) Helical wheel diagrams of the putative extended α-helical regions in DB and BD created with Heliquest.^33^ Basic residues are in blue, acidic residues are in red, polar residues are in pink and purple, small nonpolar residues are in gray, and larger nonpolar residues are in yellow.

We hypothesize that the differential impact of linkers for DB and BD relates to the potential helical orientation of residues in the hybrids. Both DesHDAP1 and BF2 have a central proline residue, followed by a C-terminal helical region. Thus, in both hybrids there is the potential for a longer putative α-helical region combining the C-terminus of the first peptide with the N-terminal region ahead of the proline in the second peptide (Figure 7A). For DB, this longer helical region would have positively charged residues aligned on the same helical face (Figure 7B). However, this is not the case in BD, with the final Lys and Arg of this region located on a hydrophobic face (Figure 7B). Thus, the presence of the proline linker in BPD would provide a structural disruption that would allow those positive charges not to be forced to the hydrophobic face of the helix. This may promote the enhanced membrane permeabilization observed for BPD compared to BD.

## Conclusion

Here we evaluated the antimicrobial activity, cytotoxicity, and mechanism of action of hybrid peptides made from the combination of BF2 and DesHDAP1. To the best of our knowledge, this is the first systematic evaluation of hybrids made from two AMPs primarily utilizing an antimicrobial mechanism involving membrane translocation. Various permutations of BF2/DesHDAP1 hybrids, including those with a variety of amino acid linkers, showed enhanced activity compared to an equimolar mixture of the two parent peptides. This is particularly striking given that hybrids made by combining DesHDAP1 or BF2 with membrane-permeabilizing peptides did not show any systematic enhancement in activity compared to mixtures of those peptides in our previous studies.^25^ It was also notable that the BF2/DesHDAP1 hybrids shifted to a more membrane-permeabilizing mechanism. In fact, it appears that the enhanced antimicrobial activity of hybrids may result from increased bacterial membrane permeabilization more than compensating for the decreased ability to access intracellular targets through membrane translocation. This is consistent with our previous observations that these and other HDAPs appear to utilize some mixture of those two mechanisms.^7^ This also implies that a design strategy that enhances permeabilization (or translocation) may only be effective if it does not cause a larger decrease in the efficacy of other mechanisms employed by that peptide, as observed in using the proline linker for BPD relative to BD.

The observation that BF2/DesHDAP1 hybrids also have no appreciable cytotoxicity against eukaryotic cells further emphasizes their value and the potential of other hybrid peptides in the design of more potent AMPs. The initial success of our approach motivates future studies focusing on hybrids made from other AMPs that significantly translocate across membranes. Although our group’s work shows that combining BF2 and DesHDAP1 together is more effective than combining either of them with membrane-permeabilizing AMPs,^25^ we suggest this is due to the specific physiochemical properties of BF2 and DesHDAP1 and does not imply a general rule that effective hybrids need to combine peptides with the same mechanism. In fact, recent work did show enhanced activity for hybrids combining temporin 1Ta/b and apidaecin 1, despite temporins causing membrane disruption and apidaecin translocating to find an intracellular target.^24,34,35^ Thus, it will be important for researchers to continue considering differing combinations of translocating AMPs in a systematic manner to best elucidate any generalizable rules to design hybrids with enhanced antibacterial activity.

## Safety

Eukaryotic HEK cells were handled following BSL2 protocols.

## Supporting Information Available

Detailed experimental methods (PDF)

## Abbreviations

AMP: antimicrobial peptide
BF2: buforin II
HDAP: histone derived antimicrobial Peptide
ROI: region of interest
PI: propidium iodide
