# Supplementary material for: Hybrids of Membrane-Translocating Antimicrobial Peptides Show Enhanced Activity through Membrane Permeabilization
Source: ACS Med Chem Lett. 2024 Oct 11;15(11):1918–24. doi: 10.1021/acsmedchemlett.4c00375 (PMC11571015; doi:10.1021/acsmedchemlett.4c00375)
Supplement: Supplementary file 1 — ml4c00375_si_001.pdf [file ml4c00375_si_001.pdf]

Supplemental Information for Trevellin et al.

Experimental Procedures

Peptide design and preparation

Sequences of the peptides used in this study are shown in Table 1. Peptides were synthesized by GenScript (Piscataway, NJ) at >95% purity determined by HPLC and dissolved in DI water. Concentrations were measured by triplicate tryptophan absorbance readings at 280 nm (ε=5700 $M^{-1}cm^{-1}$, l=1 cm) using a ThermoScientific NanoDrop 2000 (Waltham, MA). Buforin II (BF2) included an F10W mutation used in many previous studies[1-5] to allow for concentration measurements.

Bacterial preparation

Bacteria included *E. coli* (Top10 Strain, Invitrogen/ThermoFisher) containing a plasmid for ampicillin resistance (Novagen) and/or *B. subtilis* (ATCC #6051). Bacterial growth was performed in TSB (trypticase soy broth) prepared at 3% w/v (30 g of media in 1 L) from BD Difco unless otherwise noted. Overnight cultures were picked from petri dishes or frozen stock and grown for 14-16 hours at 37 °C in TSB with shaking. *E. coli* overnight cultures were incubated with 25 µg/mL ampicillin. The overnight cultures were then diluted 1:100 in 25 mL TSB and incubated at 37 °C with shaking for 2.5 h. The culture was pelleted via centrifugation at 1500×g for 10 min at room temperature and resuspended in sterile cold wash buffer (10 mM sodium phosphate buffer and 10 mM sodium chloride, pH 7.4). These resuspended bacteria were then used for radial diffusion, microbroth dilution, and propidium iodide assays.

Radial diffusion assay

Radial diffusion assays were used to determine the relative potency of each peptide. 10 mL of molten underlay (1% w/v TSB, 1% w/v agarose, 10 mM sodium chloride in 10 mM sodium phosphate buffer, pH = 7.4) was inoculated with $4x10^6$ CFU of bacteria suspended in 10 mL sterile wash buffer (10 mM sodium phosphate buffer and 10 mM sodium chloride, pH 7.4) and poured into a sterile petri dish. After the agar solidified, wells ~1 mm in diameter were formed in the agar using a glass Pasteur pipette attached to a vacuum trap. Each well was filled with 2 µL of $1x10^{-4}$ M peptide. The plate was incubated upside down for 3 h at 37 °C after which the plate was covered with 10 mL of molten overlay (6% w/v TSB, 1% w/v agarose) and incubated upside down overnight at 37 °C (18-24 h). Then, diameters of bacterial clearance were measured.

Microbroth dilution assay

Microbroth dilution assays were used to determine the minimum inhibitory concentration (MIC) of each peptide for *E. coli* and *B. subtilis* (ATCC #6051). A 1:2 serial dilution was prepared for a peptide across the top row of a 96-well plate. 100 µL of bacterial suspension (4 × 10^6 CFU/mL, diluted in sterile wash buffer, 10 mM sodium phosphate buffer and 10 mM sodium chloride, pH 7.4) and 10 µL of peptide were mixed to each well in subsequent rows of the plate. The 11 effective peptide concentrations ranged from 18.2 µM to 1.8×10^{-2} µM, with DI water used as a control, and each concentration was tested in triplicate per plate. After the bacteria and peptide mixtures were incubated in plates at 37°C for 1 hour, 100 µL of 6% w/v TSB was added to each well. For wells containing *E. coli*, ampicillin was added at a final concentration of 25 µg/ml. The plate was then incubated overnight (18-24 h) at 37°C. After incubation, OD_{600nm} were measured using the SpectraMax M3 microplate reader (Molecular Devices).

Propidium iodide (PI) uptake assay

The PI uptake assay was used to determine the relative degree of membrane permeabilization of each peptide. 3 mL of bacteria that had been resuspended in wash buffer (10 mM sodium phosphate buffer and 10 mM sodium chloride, pH 7.4) to OD_{600}=0.5 were placed in a quartz cuvette. PI was added from a 3.03 mg/mL stock solution to the cuvette to give a final concentration of 20 µg/mL. The solution equilibrated for 5 min before peptide was added to a final concentration of 2 µM. The fluorescence intensity of the solution was measured before and after the addition of the peptide using a Varian Cary Eclipse fluorescence spectrophotometer (excitation wavelength: 535 nm; emission wavelength: 617 nm). The degree of membrane permeabilization was calculated using the following equation where F_0 is the average fluorescence in the minute before peptide addition and F_5 is the average fluorescence during the time from 4-5 minutes after peptide addition:

$$\text{relative permeability} = F_5/F_0$$

Each peptide was tested with bacteria grown from at least three distinct overnight cultures.

MTS Cytotoxicity Assay

The MTS assay is a colorimetric viability assay for mammalian cells. HEK 293 cells (ATCC CRL-1573) were grown in complete growth medium (EMEM, 10% fetal bovine serum, 1% penicillin-streptomycin). Cells were cultured in a T-75 maintenance flask, fed with fresh media every 1-2 days, and passaged approximately every 5-7 days once cells reached 70-90% confluence. During passaging, HEK 293 cells were plated in a 96-well plate at 20,000 cells/well and incubated for 4-6 hours (37 °C, 95% humidity, 5% CO_2). Each column of cells (8 replicates) was then incubated overnight with 18.2 µM peptide or a control. After incubation, media was aspirated and refreshed for each well,

and 20 µL of the CellTiter 96® AQueous One Solution Reagent (Promega G3580) was added and allowed to incubate for 1-4 hours (37 °C, 95% humidity, 5% CO_2). Absorbance of each well was read at 490 nm on a Molecular Devices SpectraMax M3 microtiter plate reader.
Caution! HEK 293 cells were handled following BSL 2 protocols approved by the Wellesley College Institutional Biosafety Committee (IBC).

Spheroplast preparation and confocal microscopy

E. coli spheroplasts were prepared as described previously.[6, 7] Equal volumes (5 µL) of spheroplasts and 100 µM FITC (fluorescein isothiocyanate)-labeled peptide were pipetted on a poly-L-lysine coated glass slide, incubated with 1 µL of 0.03 mM di-8-ANEPPS for 3 minutes, and then a cover slip was added. Spheroplasts were imaged using a Leica TCS SP5 II laser scanning confocal microscope with excitation at 488 nm by an argon laser at 20% power output, 20% transmission, and emission ranges of 499–532 nm (FITC-labeled peptide) and 670–745 nm (di-8-ANEPPS). 8-bit, 512 × 512 images were collected confocally at 63X magnification, giving a section thickness of 0.8 µm (Leica Plan-Apochromat oil objectiveNA 1.40). Composite z-stack images with a 0.5 µm step size were produced by the Leica LAS AF software (Buffalo Grove, IL). Circular regions of interest (ROI) 0.3 µm in diameter were drawn on the spheroplast membrane (ROI 1), intracellular space (ROI 2), and background (ROI 3), and the fluorescence intensity of peptide was quantified in each ROI. The ratio of intracellular peptide fluorescence intensity to membrane peptide fluorescence intensity was calculated using the following equation:

$$\text{Intracellular Fluorescence/Membrane Fluorescence} = (\text{ROI2}-\text{ROI3})/(\text{ROI1}-\text{ROI3})$$

Peptide localization was defined as translocating or membrane localizing when the ratio was ≥1 and <1, respectively.

Statistical analysis

All statistical analyses were performed using SPSS version 29 (IBM). Means were compared using one-way ANOVA analyses with a Dunnett’s T3 post-hoc test that did not assume equal variances. Translocation data from confocal microscopy were compared pairwise for peptides using Fisher’s Exact test.

Supplemental Tables

Supplemental Table 1: Amino acid sequences of parent and hybrid peptides used in this study. DesHDAP1 portions of sequences are shown in bold, BF2 portions of sequences are shown in italics and linker amino acids are in red text. A^N represents hydroxyalanine. BF2 includes an F10W mutation to allow for spectroscopic measurements of peptide concentration. Physiochemical properties were computed using ProtParam (https://web.expasy.org/protparam/). Charge is predicted at pH 7 and GRAVY (Grand Average of Hydropathicity) is calculated using Kyte and Doolittle hydropathicity values.

Peptide	Amino Acid Sequence	Charge	pI	GRAVY
**DesHDAP1**	**ARDNKKTRIWPRHLQLAVRN**	**+5**	**12.01**	**-1.315**
Buforin II (BF2)	*TRSSRAGLQWPVGRVHRLLRK*	*+6*	*12.60*	*-0.814*
**DesHDAP1**-*BF2* (DB)	**ARDNKKTRIWPRHLQLAVRN**_TRSSRAGLQWPVGRVHRLLRK_	**+11**	**12.55**	**-1.059**
BF2-**DesHDAP1** (BD)	*TRSSRAGLQWPVGRVHRLLRK***ARDNKKTRIWPRHLQLAVRN**	**+11**	**12.55**	**-1.059**
**DesHDAP1**-A-*BF2* (DAB)	**ARDNKKTRIWPRHLQLAVRN**A_TRSSRAGLQWPVGRVHRLLRK_	**+11**	**12.55**	**-0.990**
BF2-A-**DesHDAP1** (BAD)	*TRSSRAGLQWPVGRVHRLLRK*A**ARDNKKTRIWPRHLQLAVRN**	**+11**	**12.55**	**-0.990**
**DesHDAP1**-P-*BF2* (DPB)	**ARDNKKTRIWPRHLQLAVRN**P_TRSSRAGLQWPVGRVHRLLRK_	**+11**	**12.55**	**-1.071**
BF2-P-**DesHDAP1** (BPD)	*TRSSRAGLQWPVGRVHRLLRK*P**ARDNKKTRIWPRHLQLAVRN**	**+11**	**12.55**	**-1.071**
**DesHDAP1**-G-*BF2* (DGB)	**ARDNKKTRIWPRHLQLAVRN**G_TRSSRAGLQWPVGRVHRLLRK_	**+11**	**12.55**	**-1.043**
BF2-G-**DesHDAP1** (BGD)	*TRSSRAGLQWPVGRVHRLLRK*G**ARDNKKTRIWPRHLQLAVRN**	**+11**	**12.55**	**-1.043**
**DesHDAP1**-A^N-*BF2* (DA^NB)	**ARDNKKTRIWPRHLQLAVRN**A^N_TRSSRAGLQWPVGRVHRLLRK_	**+11**	**12.55**	**-0.990**
BF2-A^N-**DesHDAP1** (BA^ND)	*TRSSRAGLQWPVGRVHRLLRK*A^N**ARDNKKTRIWPRHLQLAVRN**	**+11**	**12.55**	**-0.990**

Supplemental Figures

```
Histone H2A  GKQGGKTRAKAKTRSSRAGLQFPVGRVHRLLRKGNYAERVGAGAPVYLAAVLEYLTAEILELAGNAARDN
DesHDAP1                                                                      ARDN
Buforin II               TRSSRAGLQWPVGRVHRLLRK

Histone H2A  KKTRIIPRHLQLAVRNDEELNKLLGRVTIAQGGVLPNIQSVLLPKK
DesHDAP1     KKTRIWPRHLQLAVRN
```

Supplemental Figure 1: Sequence alignment of BF2 and DesHDAP1 with the sequence of Histone 2A from *Xenopus laevis*.

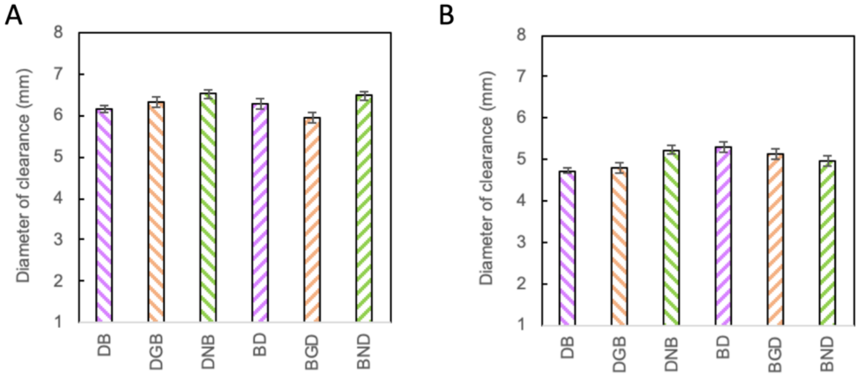

Supplemental Figure 2: (A and B) Average diameters of bacterial clearance from radial diffusion assay measurements of parent and hybrid antimicrobial peptide antibacterial activity (1x10^{-4} M) against *E. coli* (A) and *B. subtilis* (B). All averages taken over a minimum of 12 wells from at least 8 biological replicates for measurements with *E. coli* and a minimum of 12 wells from at least 3 biological replicates for *B. subtilis*. Error bars represent standard error of the mean. G refers to a glycine linker and N to a N-hydroxyalanine linker.

References

1. Kobayashi, S.; Chikushi, A.; Tougu, S.; Imura, Y.; Nishida, M.; Yano, Y.; Matsuzaki, K. Membrane translocation mechanism of the antimicrobial peptide buforin 2. *Biochemistry* **2004,** *43*, 15610-15616.
2. Kobayashi, S.; Takeshima, K.; Park, C. B.; Kim, S. C.; Matsuzaki, K. Interactions of the novel antimicrobial peptide buforin 2 with lipid bilayers: proline as a translocation promoting factor. *Biochemistry* **2000,** *39 (29)*, 8648-54.
3. Uyterhoeven, E. T.; Butler, C. H.; Ko, D.; Elmore, D. E. Investigating the nucleic acid interactions and antimicrobial mechanism of buforin II. *FEBS Lett.* **2008,** *582 (12)*, 1715-1718.
4. Wei, L.; LaBouyer, M. A.; Darling, L. E.; Elmore, D. E. Bacterial Spheroplasts as a Model for Visualizing Membrane Translocation of Antimicrobial Peptides. *Antimicrob Agents Chemother* **2016,** *60 (10)*, 6350-2.
5. Xie, Y.; Fleming, E.; Chen, J. L.; Elmore, D. E. Effect of proline position on the antimicrobial mechanism of buforin II. *Peptides* **2011,** *32 (4)*, 677-82.
6. Figueroa, D. M.; Wade, H. M.; Montales, K. P.; Elmore, D. E.; Darling, L. E. O. Production and Visualization of Bacterial Spheroplasts and Protoplasts to Characterize Antimicrobial Peptide Localization. *J Vis Exp* **2018,** *(138)*.
7. Wade, H. M.; Darling, L. E. O.; Elmore, D. E. Hybrids made from antimicrobial peptides with different mechanisms of action show enhanced membrane permeabilization. *Biochim Biophys Acta Biomembr* **2019,** *1861 (10)*, 182980.
